# Procurement of medicines to treat cancer, 2015–2020, China

**DOI:** 10.2471/BLT.22.288420

**Published:** 2022-11-02

**Authors:** Mengyuan Pan, Shuchen Hu, Jieqiong Zhang, Cheng Xiang, Zaheer-Ud-Din Babar, Caijun Yang

**Affiliations:** a Department of Pharmacy Administration and Clinical Pharmacy, School of Pharmacy, Xi’an Jiaotong University, 76 Yanta West Road, Xi'an, 710061, China.; b Department of Pharmacy, University of Huddersfield, Queensgate, England.

## Abstract

**Objective:**

To assess the procurement of medicines to treat cancer in China.

**Methods:**

We conducted a descriptive analysis of the national procurement data for 20 anti-cancer medicines in China from 2015 to 2020. We estimated the number of defined daily doses procured per year in three areas of China for essential medicines and medicines for targeted therapies. We adjusted the data by the number of cancer patients in each region for each year.

**Findings:**

Between 2015 and 2020, the number of defined daily doses per patient decreased from 40.87 to 35.86 (−12.27%) for essential medicines, while the number increased from 0.85 to 12.52 (1381.15%) for target medicines. The procurement of three out of 10 essential medicines decreased, whereas procurement of all 10 targeted medicines increased. In 2020, the eastern area procured the most essential medicines (44.98 doses per patient) and targeted medicines (16.55 doses per patient), but had the smallest relative change in procurement of both essential medicines (−22.76%) and targeted medicines (978.16%). The central area had the largest increase in procurement of both essential medicines (9.64%; from 25.25 to 27.68 doses per patient) and targeted medicines (4587.81%; from 0.23 to 10.64 doses per patient).

**Conclusion:**

Procurement of anti-cancer medicines varied across regions. Specific policies are needed at the national level to eliminate inequalities in access to these medicines. Two issues that need attention are the lower access to many essential anti-cancer medicines in some provinces and the increase in use of targeted medicines.

## Résumé

**Objectif:**

Évaluer l'approvisionnement en médicaments pour traiter le cancer en Chine.

**Méthodes:**

Nous avons réalisé une analyse descriptive des données d'approvisionnement nationales pour 20 médicaments contre le cancer en Chine, entre 2015 et 2020. Nous avons estimé le nombre de doses quotidiennes définies achetées chaque année dans trois régions du pays, aussi bien de médicaments essentiels comme de médicaments destinés à des thérapies ciblées. Nous avons ajusté ces données en tenant compte du nombre de patients atteints d'un cancer chaque année dans chacune des régions.

**Résultats:**

Entre 2015 et 2020, le nombre de doses quotidiennes définies par patient a décliné, passant de 40,87 à 35,86 (−12,27%) pour les médicaments essentiels, tandis que ce nombre a augmenté de 0,85 à 12,52 (1381,15%) pour les médicaments ciblés. L'acquisition de trois des dix médicaments essentiels a diminué; en revanche, les dix médicaments ciblés ont tous connu une hausse en matière d'approvisionnement. En 2020, c'est la région orientale qui s'est procuré le plus de médicaments essentiels (44,98 doses par patient) et ciblés (16,55 doses par patient), bien qu'elle affiche la plus petite variation relative dans l'achat de médicaments essentiels (−22,76%) et ciblés (978,16%). La région centrale, elle, a enregistré la plus importante augmentation dans l'acquisition de médicaments essentiels (9,64%; de 25,25 à 27,68 doses par patient) et ciblés (4587,81%; de 0,23 à 10,64 doses par patient).

**Conclusion:**

L'approvisionnement en médicaments contre le cancer diffère d'une région à l'autre. Des mesures spécifiques sont nécessaires à l'échelle nationale pour mettre un terme aux inégalités dans l'accès à ces traitements. Deux problèmes en particulier doivent être examinés: l'accès plus limité à de nombreux médicaments essentiels contre le cancer dans certaines provinces, ainsi que l'utilisation accrue de médicaments ciblés.

## Resumen

**Objetivo:**

Evaluar la adquisición de medicamentos para tratar el cáncer en China.

**Métodos:**

Se realizó un análisis descriptivo de los datos de adquisición nacional de 20 medicamentos contra el cáncer en China de 2015 a 2020. Se calculó la cantidad de dosis diarias definidas adquiridas por año en tres regiones de China para medicamentos esenciales y medicamentos para tratamientos dirigidos. Asimismo, se ajustaron los datos en función de la cantidad de pacientes con cáncer en cada región para cada año.

**Resultados:**

Entre 2015 y 2020, la cantidad de dosis diarias definidas por paciente disminuyó de 40,87 a 35,86 (-12,27 %) para los medicamentos esenciales, mientras que la cantidad aumentó de 0,85 a 12,52 (1381,15 %) para los medicamentos dirigidos. La adquisición de tres de los 10 medicamentos esenciales disminuyó, mientras que la adquisición de los 10 medicamentos dirigidos aumentó. En 2020, la región oriental adquirió la mayor cantidad de medicamentos esenciales (44,98 dosis por paciente) y de medicamentos dirigidos (16,55 dosis por paciente), pero tuvo el menor cambio relativo en la adquisición tanto de medicamentos esenciales (-22,76 %) como de medicamentos dirigidos (978,16 %). La región central es la que más ha aumentado la compra de medicamentos esenciales (9,64 %, de 25,25 a 27,68 dosis por paciente) y de medicamentos dirigidos (4587,81 %, de 0,23 a 10,64 dosis por paciente).

**Conclusión:**

La adquisición de medicamentos contra el cáncer varía según las regiones. Se necesitan políticas específicas a nivel nacional para eliminar las desigualdades en el acceso a estos medicamentos. Dos temas que requieren atención son el menor acceso a muchos medicamentos esenciales contra el cáncer en algunas provincias y el aumento del uso de medicamentos dirigidos.

## ملخص

**الغرض:**

تقييم شراء الأدوية لعلاج السرطان في الصين.

**الطريقة:**

قمنا بإجراء تحليل وصفي لبيانات المشتريات الوطنية لعدد 20 دواءً مقاومًا للسرطان في الصين خلال الفترة من 2015 إلى 2020. وقد قدرنا عدد الجرعات اليومية المحددة التي يتم شراؤها كل عام في ثلاث مناطق بالصين للأدوية الأساسية، وأدوية العلاجات المستهدفة. قمنا بتعديل البيانات حسب عدد مرضى السرطان في كل منطقة لكل عام.

**النتائج:**

بين عامي 2015 و2020، انخفض عدد الجرعات اليومية المحددة لكل مريض من 40.87 إلى 35.86 (−%12.27) للأدوية الأساسية، بينما ارتفع العدد من 0.85 إلى 12.52 (%1381.15) للأدوية المستهدفة. انخفض شراء ثلاثة من كل 10 أدوية أساسية، في حين زاد شراء جميع الأدوية الـ 10 المستهدفة. في عام 2020، اشترت المنطقة الشرقية الأدوية الأساسية جداً (44.98 جرعة لكل مريض)، والأدوية المستهدفة (16.55 جرعة لكل مريض)، ولكن كان لها أقل تغيير نسبي في شراء كل من الأدوية الأساسية (%22.76)، والأدوية المستهدفة (%978.16). شهدت المنطقة المركزية أكبر زيادة في شراء كل من الأدوية الأساسية (%9.64؛ من 25.25 إلى 27.68 جرعة لكل مريض)، والأدوية المستهدفة (%4587.81؛ من 0.23 إلى 10.64 جرعة لكل مريض).

**الاستنتاج:**

اختلفت مشتريات الأدوية المكافحة للسرطان عبر المناطق. هناك حاجة إلى سياسات محددة على المستوى الوطني للقضاء على حالات عدم المساواة في الحصول على هذه الأدوية. هناك مسألتان تحتاجان إلى الاهتمام، وهما الحصول المتدني على العديد من الأدوية الأساسية المكافحة للسرطان في بعض المقاطعات، والزيادة في استخدام الأدوية المستهدفة.

## 摘要

**目的:**

旨在评估中国癌症治疗药物的采购情况。

**方法:**

我们对中国 2015 年至 2020 年 20 种抗癌药物的国家采购数据进行了描述性分析。我们估计了中国三个地区每年采购的基本药物和靶向治疗药物的日规定剂量。我们根据每个地区每年的癌症患者数量对数据进行了调整。

**结果:**

在 2015 年至 2020 年期间，每名患者服用的基本药物的日规定剂量从 40.87 剂下降到 35.86 剂 (-12.27%)，靶向药物从 0.85 剂增加到 12.52 剂 (1381.15%)。十分之三的基本药物的采购量有所下降，然而，所有 10 种靶向药物的采购量均有所增加。2020 年，东部地区对基本药物（每名患者 44.98 剂）和靶向药物（每名患者 16.55 剂）的采购量最多，但对基本药物 (−22.76%) 和靶向药物 (978.16%) 的采购量的相对变化最小。中部地区对基本药物（9.64%；每名患者从 25.25 剂增加到 27.68 剂）和靶向药物（4587.81%；每名患者从 0.23 剂增加到 10.64 剂）的采购量增福最大。

**结论:**

各地区抗癌药物的采购量存在差异。需要在国家层面制定具体政策，以消除在这些药物的获得性方面的差距。需要注意两个问题，在一些省份，许多基本抗癌药物的获得性较低，并且靶向药物的使用量有所增加。

## Резюме

**Цель:**

Оценить закупки препаратов для лечения рака в Китае.

**Методы:**

В период с 2015 по 2020 год был проведен описательный анализ данных о национальных закупках 20 противораковых препаратов в Китае. Было подсчитано количество установленных суточных доз основных лекарственных средств и лекарственных препаратов для таргетной терапии, закупаемых в год в трех районах Китая. Данные были скорректированы по количеству пациентов с онкологическими заболеваниями в каждом регионе за каждый год.

**Результаты:**

В период с 2015 по 2020 год количество установленных суточных доз на одного пациента снизилось с 40,87 до 35,86 (–12,27%) для основных лекарственных средств, в то время как для лекарственных препаратов для таргетной терапии это количество увеличилось с 0,85 до 12,52 (1381,15%). Закупки трех из 10 основных лекарственных средств снизились, при этом закупки всех 10 препаратов для таргетной терапии увеличились. В 2020 году в восточном регионе было закуплено больше всего основных лекарственных средств (44,98 дозы на пациента) и лекарственных препаратов для таргетной терапии (16,55 дозы на пациента), однако относительное изменение закупок как основных лекарственных средств (–22,76%), так и препаратов для таргетной терапии (978,16%) было наименьшим. В центральном регионе наблюдался наибольший рост закупок как основных лекарственных средств (9,64%; с 25,25 до 27,68 дозы на пациента), так и лекарственных препаратов для таргетной терапии (4587,81%; с 0,23 до 10,64 дозы на пациента).

**Вывод:**

Закупки противораковых препаратов различались по регионам. Для устранения неравенства в доступе к этим лекарственным препаратам необходимы конкретные меры на национальном уровне. Две требующие внимания проблемы – это снижение доступности многих основных противораковых препаратов в некоторых провинциях и увеличение использования препаратов для таргетной терапии.

## Introduction

Cancer is a major cause of morbidity and mortality worldwide. Globally, there were 19.3 million new cancer cases and nearly 10 million cancer deaths in 2020.^1^ In 2014, cancer deaths accounted for 22.4% (2.205 million/9.846 million) of the total deaths in China.^2^ Cancer caused a total loss of 233.5 million disability-adjusted life-years worldwide in 2017;^3^ of these, about 62.9 million were in China.^4^ During the past decade, the annual increases of cancer incidence and mortality in China have been about 3.9% and 2.5%, respectively.^5^

Access to anti-cancer medicines, in suitable dosage forms and at reasonable prices, is essential to improve health outcomes for cancer patients. In 2009, the Chinese government began establishing a national essential medicines system.^6^ The first national essential medicines list was issued in 2009 and updated in 2012 and 2018.^7^^–^^9^ The 2009 list contained 307 medicines, none of which were anti-cancer medicines, whereas the 2012 list contained 520 medicines, 26 of which were anti-cancer medicines, for use in traditional chemotherapy and endocrine therapy. New medicines for targeted therapies were introduced in the early 2000s and widely used in cancer treatment. Targeted medicines have been included on the National Reimbursement Drug List since 2017 and six targeted medicines were included in the 2018 national essential medicines list. However, targeted medicines do not always deliver clinically meaningful benefit and are more costly than traditional chemotherapy medicines.^10^^–^^13^

With increasing incidence of, and mortality from, cancer in China, improving access to anti-cancer medicines is needed. However, only limited national data are available about access to different kinds of anti-cancer medicines in China. Most studies on this issue have concentrated on a small number of hospitals in certain areas, and none have compared the use of different classes of anti-cancer medicines.^14^^–^^16^

To fill this research gap, we analysed data on procurement of anti-cancer medicines in different regions of China from 2015 to 2020. We included both essential medicines and high-cost targeted therapies, which we classified as targeted medicines.

## Methods

### Medicine selection

We considered the five cancers with the highest incidence in China: lung, gastric, colorectal, liver and breast cancers.^17^ We selected the commonly used medicines for treating these cancers, as recommended in cancer diagnosis and treatment guidelines from the Medical Administration and Hospital Authority.^18^^–^^20^ Medicines were selected if they met all three criteria: (i) an anti-cancer medicine based on the anatomical therapeutic chemical drug classification method or the drug database in China; (ii) on the 2012 national essential medicines list^8^ or could be determined as a targeted medicine through the mode of drug action; and (iii) on the market in China before 2015.

### Data sources

We extracted monthly data on procurement of anti-cancer medicines from 2015 to 2020 from 31 provincial National Centralized Drug Procurement service centres. The data included the generic name, chemical substance name, dosage form, strength and quantity of the medicine. We excluded two medicines (rapamycin and cyclophosphamide) and three provinces (Heilongjiang, Ningxia and Tibet) because data were not available.

The defined daily dose of a medicine is the assumed average maintenance dose per day for the medicine used for its main indication in adults; defined daily dose is commonly used as a unit for measuring the level of use of a medicine in a population. Defined daily doses are assigned to medicines by the World Health Organization (WHO) Collaborating Centre for Drug Statistics Methodology, but have not been assigned to anti-cancer medicines by WHO because of large differences in individual treatment plans.^21^ Therefore, we used defined daily dose values provided by the German Federal Institute for Drugs and Medical Devices.^22^ For most medicines, these values are similar to the daily dose for the main treatment recommended in the product information for the medicine. Where we could not obtain a defined daily dose for a medicine, we used the daily dose for the main treatment in the medicine’s product information as a reference. We excluded methotrexate and everolimus because they both have multiple anatomical therapeutic chemical codes and multiple defined daily doses. We finally included 20 anti-cancer medicines, comprising 10 essential medicines and 10 non-essential targeted medicines (Table 1).

**Table 1:** Classification and defined daily dose of included anti-cancer medicines

Medicine	Defined daily dose (mg)
**Essential **	
Arsenic trioxide	6.19
Calcium folinate	60
Carboplatin	25
Cisplatin	6.75
Doxorubicin	5
Etoposide	50(oral) 25.00 (parenteral)
Fluorouracil	150 (oral) 100 (parenteral)
Oxaliplatin	11
Paclitaxel	15
Tamoxifen	20
**Targeted **	
Kinase inhibitor	
Apatinib	850
Crizotinib	500
Erlotinib	150
Gefitinib	250
Icotinib	125
Lapatinib	1250
Sorafenib	800
Monoclonal antibody	
Bevacizumab	45
Cetuximab	65
Trastuzumab	20

## Analysis

The 28 provinces included were divided into three regions for analysis: eastern (Beijing, Fujian, Guangdong, Hainan, Hebei, Jiangsu, Liaoning, Shandong, Shanghai, Tianjin and Zhejiang), central (Anhui, Henan, Hubei, Hunan, Jiangxi, Jilin and Shanxi) and western (Chongqing, Gansu, Guangxi, Guizhou, Inner Mongolia, Qinghai, Shaanxi, Sichuan, Xinjiang and Yunnan).

We divided the total quantity of each medicine purchased by its defined daily dose to estimate the number of defined daily doses procured per year. To obtain the defined daily doses procured per patient, we divided these data by the number of cancer patients in each region for each year (Table 2). Detailed calculation process is shown in the data repository.^25^ We also categorized the 10 targeted medicines as monoclonal antibodies or kinase inhibitors to explore possible patterns in procurement for these different types of targeted medicines.

**Table 2:** Cancer incidence and number of cancer patients, China, 2015–2020

Characteristic	Year					
	2015	2016	2017	2018	2019	2020
**National**						
Population in 10 000^a^	133 554	134 505	135 326	135 919	136 472	136 755
Incidence per 100 000 population^b^	230.33	230.05	234.41	239.83	244.75	243.59
**Eastern region** ^c^						
Population in 10 000^a^	58 541	59 114	59 595	59 997	60 351	60 690
No. of cancer patients^d^	1 402 988	1 398 603	1 437 119	1 478 968	1 512 815	1 509 807
**Central region** ^c^						
Population in 10 000^a^	38 794	38 898	38 970	38 991	39 021	38 844
No. of cancer patients^d^	904 283	896 887	927 961	952 247	973 704	972 895
**Western region** ^c^						
Population in 10 000^a^	36 219	36 493	36 761	36 931	37 100	37 221
No. of cancer patients^d^	768 870	798 791	807 084	828 594	853 572	848 582

^a^ Data source: National Bureau of Statistics of China.^23^

^b^ Data source: Global Burden of Disease study, adjusted by standard age.^24^ Detailed calculation process is shown in the data repository.^25^

^c^ Provinces included in the eastern region are: Beijing, Fujian, Guangdong, Hainan, Hebei, Jiangsu, Liaoning, Shandong, Shanghai, Tianjin, Zhejiang; in the central region are: Anhui, Henan, Hubei, Hunan, Jiangxi, Jilin, Shanxi; in the western region are: Chongqing, Gansu, Guangxi, Guizhou, Inner Mongolia, Qinghai, Shaanxi, Sichuan, Xinjiang, Yunnan.

^d^ Detailed calculation process is shown in the data repository.^25^

We used the Wilcoxon signed rank test to identify whether changes in the use of essential and targeted medicines in provinces between 2015 and 2020 were significant. The Pearson correlation coefficient was used to measure the relationship between absolute change in defined daily dose per patient from 2015 to 2020 and per capita gross domestic product (GDP) in 2020 in each province.^26^

## Results

**Fig. 1:**
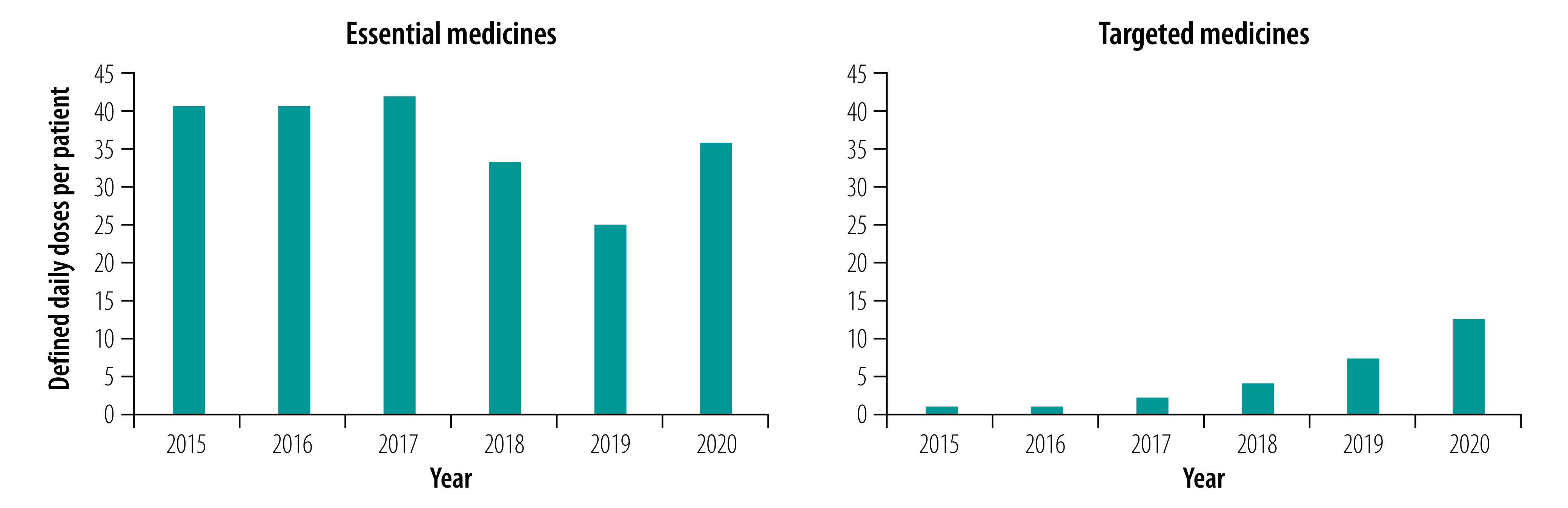
Procurement of medicines to treat cancer, per patient, China, 2015–2020

Note: Provinces included are presented in Table 6

Overall, procurement of essential medicines was much higher than that of targeted medicines in all years (Fig. 1). The eastern area procured more essential and targeted medicines per patient than the two other areas (Table 3). In 2020, the eastern area procured 44.98 defined daily doses per patient of essential medicines, while the central and western areas procured 27.68 and 28.99 doses, respectively. For targeted medicine, the eastern area procured 16.55 doses, compared with 10.64 doses in central and 7.52 doses in western areas. 

**Table 3:** Procurement of medicines to treat cancer, by area, China, 2015 and 2020

Characteristic	Eastern	Central	Western	Nationwide
**Estimated no. of patients**				
2015	1 402 988	904 283	768 870	3 076 141
2020	1 509 807	972 895	848 582	3 331 283
Absolute change	106 819	68 611	79 712	255 143
Relative change, %	7.61	7.59	10.37	8.29
**Essential medicines, defined daily doses per capita**				
2015	58.24	25.25	27.56	40.87
2020	44.98	27.68	28.99	35.86
Absolute change	−13.25	2.43	1.43	−5.01
Relative change, %	−22.76	9.64	5.20	−12.27
**Targeted medicines, defined daily doses per patient**				
2015	1.53	0.23	0.31	0.85
2020	16.55	10.64	7.52	12.52
Absolute change	15.01	10.41	7.20	11.67
Relative change, %	978.16	4587.81	2289.56	1381.15

Note: Inconsistencies may arise in some values because we calculated absolute and relative changes using real data and not the estimated procured defined daily doses per patient presented in the table for years 2015 and 2020. Detailed calculation process is shown in the data repository.^25^

**Fig. 2:**
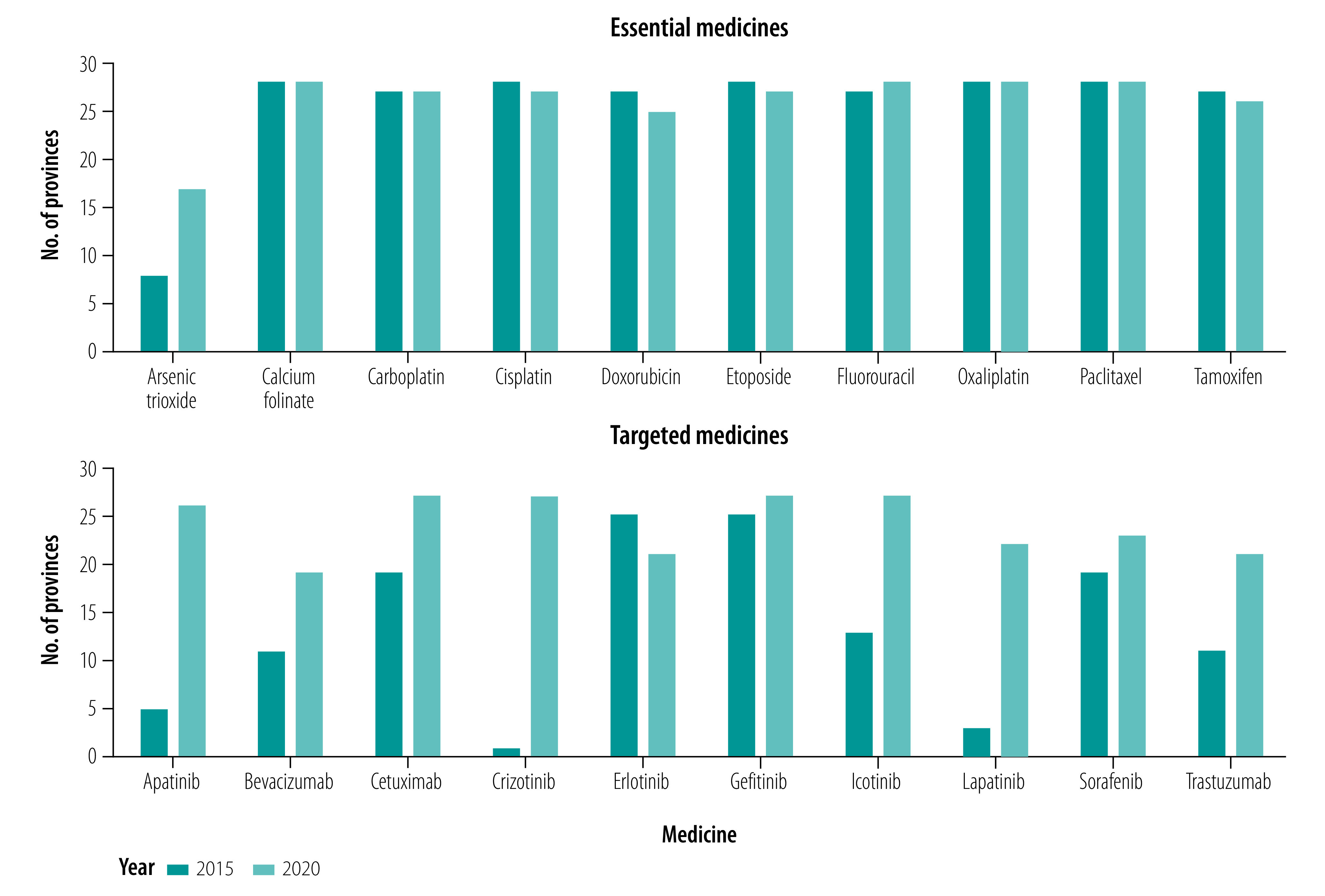
Number of provinces procuring medicines to treat cancer, China, 2015 and 2020

Note: Provinces included are presented in Table 6.

The number of provinces procuring essential medicines, averaged across the different medicines, was 26 in both 2015 (range: 8–28) and 2020 (range: 17–28). The average number of provinces procuring targeted medicines was 13 (range: 1–25) in 2015 and 24 (range: 19–27) in 2020 (Fig. 2).

For essential medicines, five out of 10 were available in all 28 provinces in 2015. In 2020, only four medicines were procured by all 28 provinces. Arsenic trioxide was procured by the lowest number of provinces in both years; however, the number of provinces procuring it increased the most, from eight provinces in 2015 to 17 provinces in 2020. Overall, doxorubicin, cisplatin, tamoxifen and etoposide were procured by fewer provinces in 2020 compared with 2015, and fluorouracil and arsenic trioxide were procured by more provinces in 2020 compared with 2015 (Fig. 2).

The most widely procured targeted medicines in 2015 were erlotinib and gefitinib, which were procured by 25 provinces. In 2020, the number of provinces procuring gefitinib had increased to 27, whereas the number of provinces procuring erlotinib had decreased to 21. Only one province procured crizotinib in 2015, but in 2020, 27 provinces were procuring the medicine. Overall, nearly all targeted medicines were procured by more provinces in 2020 than in 2015; only erlotinib was procured by fewer provinces (Fig. 2).

### Changes in procurement

#### Nationwide

The procurement of targeted medicines in China increased each year from 0.85 defined daily doses per patient in 2015 to 12.52 defined daily doses per patient in 2020. For essential medicines, 40.87 defined daily doses per patient were procured in 2015. While the procurement began to decline in 2018, it increased again in 2020 to 35.86 defined daily doses per patient (Fig. 1; Table 3). 

The relative change in the number of defined daily doses per patient was much larger for targeted medicines than for essential medicines (1381.15% versus −12.27%; Table 3). Seven essential medicines showed increases in the procurement, with the greatest increase for fluorouracil (3.80 defined daily doses per patient). Of the three medicines decreasing in procurement, tamoxifen decreased the most (−10.60 defined daily doses per patient) (Table 4).

**Table 4:** Changes in procurement of essential medicines to treat cancer, China, 2015 and 2020

Medicine	Eastern area					Central area					Western area					Nationwide			
	Defined daily doses per patient		Absolute change	Rank		Defined daily doses per patient		Absolute change	Rank		Defined daily doses per patient		Absolute change	Rank		Defined daily doses per patient		Absolute change	Rank
	Year 2015	Year 2020				Year 2015	Year 2020				Year 2015	Year 2020				Year 2015	Year 2020		
Arsenic trioxide	0.05	0.06	0.00	5		0.00	0.05	0.04	5		0.06	0.04	−0.01	7		0.04	0.05	0.01	7
Calcium folinate	2.68	1.66	−1.03	8		1.73	0.80	−0.93	9		1.55	1.37	−0.19	9		2.12	1.33	−0.79	9
Carboplatin	3.55	5.47	1.92	2		1.26	3.02	1.76	2		1.19	3.00	1.81	1		2.29	4.12	1.84	2
Cisplatin	5.42	5.06	−0.36	7		3.28	3.74	0.46	4		3.39	3.95	0.57	4		4.28	4.40	0.11	5
Doxorubicin	0.31	0.52	0.21	4		0.47	0.27	−0.21	6		0.34	0.27	−0.08	8		0.36	0.38	0.02	6
Etoposide	2.30	3.42	1.13	3		1.51	1.28	−0.23	7		0.96	1.34	0.38	5		1.73	2.27	0.54	4
Fluorouracil	4.80	11.14	6.34	1		2.87	4.71	1.83	1		3.58	5.11	1.53	3		3.93	7.72	3.80	1
Oxaliplatin	6.99	6.70	−0.29	6		3.59	4.90	1.31	3		3.18	4.94	1.75	2		5.04	5.72	0.68	3
Paclitaxel	2.84	1.71	−1.14	9		1.85	1.19	−0.65	8		1.83	2.16	0.33	6		2.30	1.67	−0.63	8
Tamoxifen	29.29	9.26	−20.03	10		8.69	7.72	−0.96	10		11.48	6.81	−4.66	10		18.78	8.19	−10.60	10

Note: Inconsistencies may arise in some values because we calculated absolute and relative changes using real data and not the estimated procured defined daily doses per patient presented in the table for years 2015 and 2020. Detailed calculation process is shown in the data repository.^25^

#### Areas

**Fig. 3:**
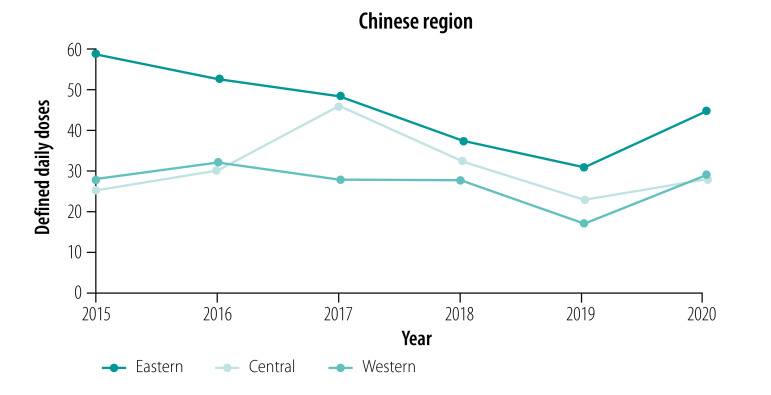
Defined daily doses per patient of essential medicines to treat cancer, by area, China, 2015–2020

**Fig. 4:**
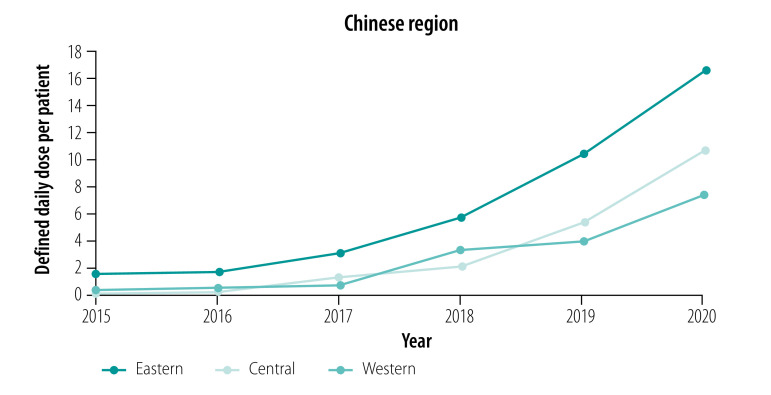
Defined daily doses per patient of targeted medicines to treat cancer, by area, China, 2015–2020

In the eastern and western areas, the number of defined daily doses per patient for essential medicines declined from 2015 to 2019; in the central area the procurement initially increased, but started declining in 2018 and again in 2019. Between 2019 and 2020 the procurement of essential medicines per patient increased in all areas (Fig. 3). For targeted medicines, the number of defined daily doses per patient showed an upward trend in all areas (Fig. 4).

While procurement of essential medicines (measured as defined daily doses per patient) decreased from 58.24 in 2015 to 44.98 in 2020 in the eastern area, it slightly increased in the central area (from 25.25 to 27.68) and western area (from 27.56 to 28.99). The eastern area had the smallest relative change in the number of defined daily doses per patient from 2015 to 2020 (−22.76% for essential medicines and 978.16% for targeted medicines). The central area had the largest relative change for essential medicines (9.64%) and targeted medicines (4587.81%) (Table 3).

In the central and eastern areas, fluorouracil showed the largest growth (1.83 and 6.34 defined daily doses per patient, respectively) in procurement of essential medicines. Five of the 10 essential medicines showed decreases in these areas. In the western area, carboplatin showed the largest growth (1.81 defined daily doses per patient) of essential medicines. Four of the 10 essential medicines showed decreases (Table 4).

All targeted medicines showed increases in procurement in all areas. The increase was greatest for icotinib (3.93 defined daily doses per patient nationally) and smallest for lapatinib (0.03 defined daily doses per patient nationally; Table 5). There was no obvious pattern in the changes in procurement of different categories of targeted medicines (further details in data repository).^25^

**Table 5:** Changes in procurement of targeted medicines to treat cancer, China, 2015 and 2020

Medicine	Eastern area					Central area					Western area					Nationwide			
	Defined daily doses per patient		Absolute change	Rank		Defined daily doses per patient		Absolute change	Rank		Defined daily doses per patient		Absolute change	Rank		Defined daily doses per patient		Absolute change	Rank
	Year 2015	Year 2020				Year 2015	Year 2020				Year 2015	Year 2020				Year 2015	Year 2020		
Apatinib	0.00	0.34	0.34	8		0.00	0.71	0.71	4		0.00	0.16	0.16	7		0.00	0.40	0.40	5
Bevacizumab	0.03	0.65	0.63	4		0.01	0.41	0.40	5		0.00	0.20	0.20	5		0.02	0.47	0.45	4
Cetuximab	0.03	0.46	0.44	6		0.00	0.12	0.12	8		0.00	0.08	0.07	8		0.01	0.26	0.25	8
Crizotinib	0.00	0.47	0.47	5		0.00	0.36	0.36	6		0.00	0.18	0.18	6		0.00	0.37	0.37	6
Erlotinib	0.12	0.31	0.20	9		0.03	0.11	0.08	9		0.07	0.13	0.06	9		0.08	0.21	0.13	9
Gefitinib	0.29	3.79	3.50	3		0.10	2.46	2.36	3		0.16	2.15	1.99	2		0.20	2.98	2.78	3
Icotinib	0.95	5.96	5.01	1		0.07	3.31	3.23	1		0.05	2.88	2.84	1		0.47	4.40	3.93	1
Lapatinib	0.00	0.04	0.04	10		0.00	0.01	0.01	10		0.00	0.03	0.03	10		0.00	0.03	0.03	10
Sorafenib	0.04	0.46	0.42	7		0.01	0.32	0.31	7		0.01	0.30	0.30	4		0.02	0.38	0.36	7
Trastuzumab	0.08	4.05	3.97	2		0.01	2.83	2.83	2		0.03	1.39	1.36	3		0.05	3.02	2.97	2

Note: Inconsistencies may arise in some values because we calculated absolute and relative changes using real data and not the estimated procured defined daily doses per patient presented in the table for years 2015 and 2020. Detailed calculation process is shown in the data repository.^25^

#### Provinces

For essential medicines, four provinces (Fujian, Hainan, Shanghai and Tianjin) out of 11 in the eastern area had an absolute increase in procurement from 2015 to 2020; Shanghai showed the largest increase among all 28 provinces (216.41 defined daily doses per patient). Out of seven provinces in the central area, three showed an increase (Anhui, Hubei and Shanxi); Anhui contributed the most (27.37 defined daily doses per patient). Among the 10 western provinces, five (Chongqing, Guizhou, Inner Mongolia, Shaanxi and Sichuan) showed an increase. Inner Mongolia contributed the most (17.68 defined daily doses per patient) (Table 6).

**Table 6:** Changes in procurement of medicines to treat cancer, by province, China, 2015 and 2020

Province	Essential medicine					Targeted medicine			
	Defined daily doses per patient		Absolute change	Rank		Defined daily doses per patient		Absolute change	Rank
	Year 2015	Year 2020				Year 2015	Year 2020		
**Eastern area**									
Beijing	104.97	93.21	−11.76	7		7.73	51.67	43.94	3
Fujian	5.46	39.71	34.25	3		0.00	16.74	16.74	5
Guangdong	42.04	29.86	−12.18	8		0.52	15.35	14.83	6
Hainan	3.78	31.67	27.89	4		0.89	14.13	13.24	7
Hebei	22.45	0.04	−22.42	10		0.12	4.48	4.36	11
Jiangsu	60.40	56.28	−4.12	5		0.18	9.42	9.24	9
Liaoning	35.35	29.58	−5.77	6		0.77	11.94	11.17	8
Shandong	118.96	15.62	−103.34	11		0.75	5.30	4.54	10
Shanghai	77.48	293.89	216.41	1		6.84	65.07	58.23	1
Tianjin	61.73	112.37	50.64	2		0.22	50.53	50.31	2
Zhejiang	65.17	46.81	−18.36	9		5.97	25.28	19.31	4
**Central area**									
Anhui	11.06	38.43	27.37	1		0.00	16.07	16.07	2
Henan	54.58	41.54	−13.04	7		0.66	23.57	22.92	1
Hubei	0.30	14.55	14.24	3		0.00	7.13	7.13	3
Hunan	29.58	17.36	−12.22	6		0.02	0.53	0.51	7
Jiangxi	32.17	26.76	−5.41	5		0.04	4.63	4.59	4
Jilin	15.81	14.25	−1.56	4		0.81	2.99	2.18	6
Shanxi	0.13	21.11	20.97	2		0.00	2.33	2.33	5
**Western area**									
Chongqing	23.70	37.48	13.78	2		0.22	16.76	16.54	2
Gansu	25.68	12.59	−13.09	9		0.03	3.55	3.52	8
Guangxi	35.06	12.76	−22.30	10		0.28	1.14	0.86	9
Guizhou	7.43	18.68	11.25	3		0.05	7.30	7.25	5
Inner Mongolia	29.12	46.80	17.68	1		0.74	22.01	21.27	1
Qinghai	24.55	16.64	−7.91	7		0.98	0.32	−0.66	10
Shaanxi	27.15	30.26	3.10	5		0.29	10.74	10.45	3
Sichuan	36.18	47.26	11.08	4		0.01	4.11	4.10	7
Xinjiang	26.17	16.08	−10.09	8		2.30	11.57	9.26	4
Yunnan	24.86	23.74	−1.11	6		0.01	4.93	4.92	6

Note: Inconsistencies may arise in some values because we calculated absolute and relative changes using real data and not the estimated procured defined daily doses per patient presented in the table for years 2015 and 2020. Detailed calculation process is shown in the data repository.^25^

All provinces procured more targeted medicines in 2020 than in 2015, except Qinghai in the western area (−0.66 defined daily doses per patient). Shanghai (58.23 defined daily doses per patient), Henan (22.92 defined daily doses per patient) and Inner Mongolia (21.27 defined daily doses per patient) accounted for the largest increases in the eastern, central and western areas, respectively (Table 6).

For all 28 provinces, changes in procurement during the study period were significant for targeted medicines (*P*-value: 0.000), but not significant for essential medicines (*P*-value: 0.716).

**Fig. 5:**
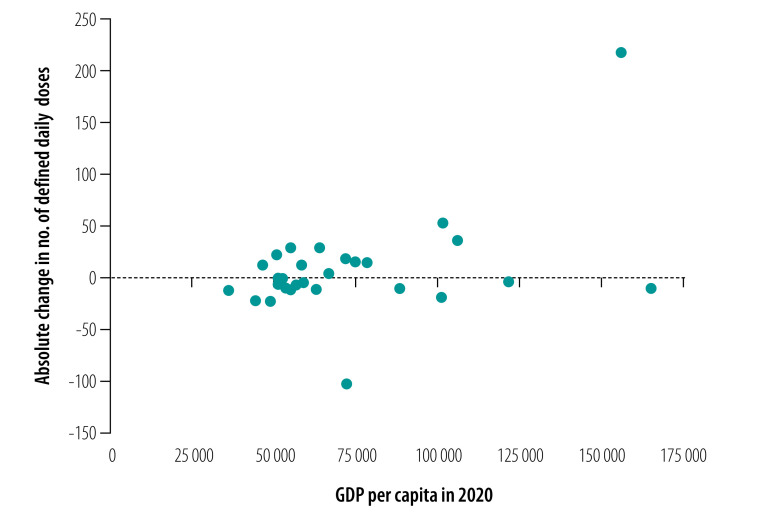
Relationship between defined daily doses per patient of essential medicines to treat cancer and GDP per capita, by Chinese province, 2020

**Fig. 6:**
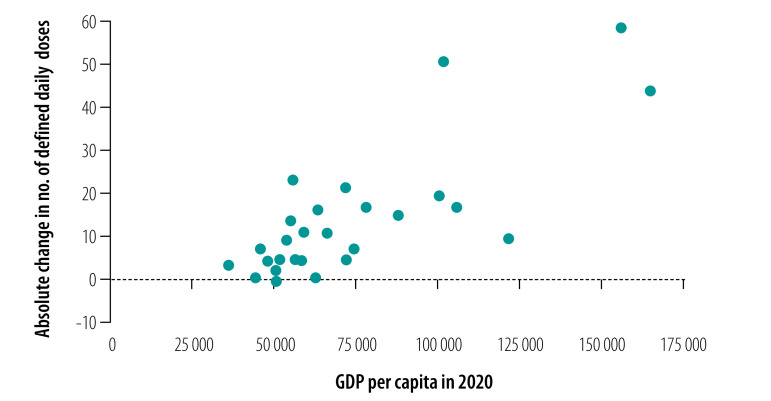
Relationship between defined daily doses per patient of targeted medicines to treat cancer and GDP per capita, by Chinese province, 2020

Fig. 5 and Fig. 6 show the relationship between the absolute change in the number of defined daily doses per patient in each province and GDP per capita in 2020. Overall, provinces with higher GDP had a greater change in the procurement of essential medicines (coefficient: 0.458; *P*-value: 0.014) and targeted medicines (coefficient: 0.788; *P*-value: 0.000).

GDP: gross domestic product.

Note: Provinces included are presented in Table 6.

GDP: gross domestic product.

Note: Provinces included are presented in Table 6.

## Discussion

Here we show that essential medicines had a better provincial coverage and higher procurement than targeted medicines. However, the total procurement of essential medicines decreased, whereas the procurement of targeted medicines increased over the study period. Nationally, procurement of nearly one third of the essential medicines included in this study decreased, whereas procurement of all the targeted medicines increased.

The higher procurement of essential medicines can be attributed to China’s reimbursement policy and drug prices. When establishing the national essential medicines system, each provincial government set specific targets for health-care institutions in acquiring and using all types of essential medicines. For example, the secondary hospital in Shaanxi Province had a target of at least 40% of all medicines sales volume being for essential medicines.^27^ All essential medicines are included as class A drugs, which are drugs that are fully reimbursed, in the National Reimbursement Drug List (2009),^28^ whereas the targeted medicines in our study were only included as class B drugs (partial reimbursement) after 2017 (eight medicines) or 2018 (two medicines).^29^^,^^30^

In addition, targeted medicines are much more expensive than essential medicines,^31^ and their procurement will impose an economic burden on patients. 

On the other hand, targeted medicines showed a much greater relative change in procurement than essential medicines. Although the level of reimbursement is lower for targeted medicines than for essential medicines, including targeted medicines in medical insurance can promote greater use.^32^ Any impact on the affordability of medical insurance should be continuously monitored.

To control the government’s expenditure, risk-sharing arrangements can be introduced when these innovative medicines are included on the National Reimbursement Drug List, and real-world evidence can be used to monitor the medicines’ cost–effectiveness.^33^^,^^34^ If a targeted medicine has no meaningful clinical benefits compared with essential medicines, clinical guidelines should recommend use of the latter over the former. Currently, the Chinese health-care system is not well equipped to support successful implementation of risk-sharing arrangements. Although China has registries and databases for patient-level and drug utilization data, the patient-level data are limited, and the quality of the data is insufficient to support risk-sharing arrangements.^35^ However, with the implementation of the diagnosis-related groups system (a system that groups patients with similar diagnoses who require similar hospital services), which is being piloted in China, and further investment in medical databases, risk-sharing arrangements are possible strategy choices for Chinese policy-makers in future.

Overall, this study found that the procurement of anti-cancer medicines varied across areas. The eastern area procured more essential and targeted medicines than other areas. This finding may be the result of differences in regional economic conditions and regional medical insurance policies. In China, the eastern area is relatively economically developed and has better access to health resources, compared with the other areas.^36^ China’s current basic medical insurance is based on the prefecture-level and county-level city as the basic pooling unit, meaning that cities formulate specific reimbursement policies. Economically developed areas tend to offer higher levels of protection.^37^ Therefore, the availability and affordability of anti-cancer medicines are higher in high-income areas than in low-income areas.^38^ Our results show a huge disparity in procurement of anti-cancer medicines, which may lead to inequalities in health outcomes in China.

Procurement of essential medicines decreased in the eastern area from 2015 to 2020, but the absolute increase in procurement of targeted medicines in the eastern area was the highest among the three areas. To some extent, this finding can be explained by patients in the more developed area tending to use more high-priced targeted medicines.^39^ However, the central and western areas had a higher relative increase in procurement of targeted medicines than the eastern area. Considering the high price of targeted medicines, the increased economic burden on cancer patients in the central and western areas is a concern.

We observed an increase in the procurement of all targeted medicines studied at the national level. These results are similar to a published study that showed that sales from 2007 to 2017 across all types of anti-cancer therapies increased in China.^40^ In contrast, our study showed that procurement of one third of the essential medicines decreased. At the provincial level, we also found that the procurement of essential medicines decreased in more than half the provinces, and the procurement of targeted medicines decreased only slightly in one province. The combined results suggest large disparities in access among provinces, including less access to some essential medicines by the populations of some provinces.

In our analysis of targeted medicines, we found that the procurement of icotinib increased substantially in all regions during the study period. This finding may be related to national negotiations that led to a 54% decrease in the price of icotinib in 2016.^41^ The targeted medicine gefitinib also decreased in price as a result of the negotiations, and its procurement also increased substantially after 2016. Researchers who analysed the impact of national negotiations on icotinib and gefitinib found similar results.^42^ However, these national negotiations might have had a negative impact on equity of access to targeted anti-cancer medicines, since a study showed that the eastern provinces benefited more from the negotiations than central and western provinces.^43^ The reason behind this difference is uncertain.

The Chinese government could introduce specific policies to eliminate inequalities in access to anti-cancer medicines across areas. Providing full coverage for some of the essential anti-cancer medicines for both inpatients and outpatients and free early cancer screening programmes in these less developed areas might be feasible solutions. Full coverage for both inpatients and outpatients has been adopted for medicines used to treat hypertension and diabetes in some pilot cities and has proved effective in reducing costs and promoting medicine use.^44^ Full coverage for essential anti-cancer medicines may promote their use and reduce demand for expensive targeted medicines, especially when the two therapies lead to similar clinical benefits. Providing organized free screening services for high-risk populations and cancers can reduce the cancer burden^45^ and the demand for anti-cancer medicines.

Our study has several limitations. First, we used procurement data for anti-cancer medicines in different provinces. Although procurement data can reflect clinical demand over time, they do not reflect actual clinical use or patient access. To minimize this potential bias, we used annual data to analyse changes in the overall procurement of different medicines nationwide. However, procurement data may differ from actual use if the number of patients presenting for treatment differs between years. Second, we used data on incidence of all five cancers in each region, rather than incidence of the separate cancers. The incidence data for 2015–2019 were estimated by the Global Burden of Disease study.^24^ We did not consider cross-regional cancer treatment, which is rare. These issues mean that we could only make crude estimates of the number of cancer patients per year in each area. Third, we considered only 20 medicines; other medicines for the five cancers were not included. Although these 20 medicines are recommended by the National Health Commission, physicians in some areas may prefer to use other medicines. Fourth, we used the defined daily doses provided by the German Federal Institute for Drugs and Medical Devices to standardize the measure of medicine use. Although most of the values for the included medicines were similar to the daily dose for the main indication recommended in the medicines’ product information, they have not been validated by WHO. Finally, we used the corresponding regional incidence when calculating the absolute change in medicine procurement in each province. Therefore, variations in procurement of anti-cancer medicines among provinces may partly be attributed to variation in cancer incidence among provinces. Furthermore, some medicines can be procured for other types of cancers.

This study revealed trends and disparities in the procurement of anti-cancer medicines among different areas of China. While the decrease in the procurement of essential anti-cancer medicines in many provinces suggest a reduced access by patients, the increase in the procurement of targeted medicines in almost all provinces might impose an economic burden to the patients. These two issues need attention by Chinese government. Specific policies can be implemented to improve the access to essential anti-cancer medicines and monitor the rapid growth in the use of targeted medicines. 
